# The evolution of the field of Health Policy and Systems Research and outstanding challenges

**DOI:** 10.1186/s12961-018-0317-x

**Published:** 2018-05-23

**Authors:** Sara Bennett, Julio Frenk, Anne Mills

**Affiliations:** 1 Department of International Health, Johns Hopkins Bloomberg School of Public Health, Baltimore, MD 21205 United States of America; 2 University of Miami, 230 Ashe Building, 1252 Memorial Drive, Coral Gables, Florida, 33146 United States of America; 3 London School of Hygiene and Tropical Medicine, Keppel St, London, WC1E 7HT United Kingdom

## Abstract

**Background:**

We provide a historical analysis of the evolution of the field of health policy and systems research (HPSR) since 1996. In the mid-1990s, three main challenges affected HPSR, namely (1) fragmentation and lack of a single agreed definition of the field; (2) ongoing dominance of biomedical and clinical research; and (3) lack of demand for HPSR. Cross-cutting all these challenges was the problem of relatively limited capacity to undertake high quality HPSR. Our discussion analyses how these problems were addressed so as to facilitate growth and enhanced recognition of the field.

**Discussion:**

HPSR has benefitted significantly from increased recognition of the importance of strong health systems to health outcomes, particularly those linked to the Millennium Development Goals. In addition to this, some of the challenges described above have been addressed through (1) sustained advocacy for the importance of HPSR, (2) efforts to clarify the content and focus of the field, and (3) growing appreciation of and efforts to engage health practitioners and policy-makers in HPSR. While advocacy for the field of HPSR was initially fragmented, since the late 1990s there has been a consistent flow of focusing events and publications that have served to enhance the profile and understanding of the field. There have also been multiple efforts to establish greater coherence within the field, for example, interrogating the distinctions between health services research and health systems research, and how critical the “P” for policy is to HPSR. Finally, HPSR has developed at the same time as growing interest in evidence-informed policy and, more recently, implementation science, which have served to underscore the relevance and utility of HPSR to policy- and decision-makers.

**Conclusions:**

During the past two decades, the field of HPSR has developed significantly, leading to enhanced clarity about its purpose, activity levels and utility. Several challenges remain that will need to be addressed in the decades ahead.

## Background

The field of health policy and systems research (HPSR) has demonstrated a remarkable maturation over the past 20 years, wherein the level of funding, the number of publications and the number of researchers engaged in HPSR have all grown substantially [1]. We seek to explain why and how this growth has occurred. In 1996, WHO published the volume *Health Policy and Systems Development: An Agenda for Research* [2], which laid the foundation for the establishment of the Alliance for HPSR in 1999; therefore, we use this date, 1996, as the starting point for our analysis.

As for many new fields of endeavour in the field of development, the evolution of HPSR reflects a constant back and forth between individual country interests and aspirations, on the one hand, and global level processes, on the other. Given the diversity of country experiences and responses, global level processes are often more visible and recognisable, and indeed they are the primary focus of this paper. However, this focus means that we inevitably provide only a partial view of the forces that have shaped HPSR in individual countries.

### 1996: Three core challenges for HPSR

Three principal challenges to the growth of the field of HPSR existed in the mid-1990s. First, the field of HPSR was just emerging. While several international and national centres focused on different aspects of health systems, including their financing and organisation, there was no common understanding of how various components of a health system, e.g. health financing, the private sector or community health systems, might fit together. The problem of lack of definition of the field was further exacerbated by confusion between the terms ‘health systems research’ and ‘health services research’. The latter formed a relatively well accepted and supported field of study in high-income countries that appeared to overlap with, but also differ from, health systems research, which was primarily discussed with reference to low- and middle-income countries (LMICs) [3]. While health services research, at the time, focused primarily on micro- and meso-level questions about the interaction between patients, providers and service delivery organisations, health systems research typically focused on more macro-level questions concerned with the organisation of health systems as a whole.

Second, health research funding had a strong bias towards biomedical and clinical research, as highlighted in the 1990 report of the Commission on Health Research for Development [4]. The Commission’s report drew attention to particularly under-funded areas of research, underlining the neglect of “*policy and social science, and management research*” as well as “*problems not classified as diseases, such as health information systems, costs and financing, and the wasteful misuse of drugs*”. The existing bias towards biomedical and clinical research had broader ramifications, particularly with respect to the development of research capacity. Given the context-specific nature of much HPSR, it depends on the existence of capacity in every country and preferably at subnational levels too, whereas biomedical and clinical research can rely more on regional centres of excellence. Thus, the dominance of a biomedical and clinical research paradigm also contributed to severe imbalances in research capacity identified in the 1990 Commission report [4] and in later reports by both the Council on Health Research for Development [5] and the Global Forum on Health Research [6].

Finally, a critical challenge for HPSR during the mid-1990s was a lack of demand for evidence to inform decision-making about health systems strengthening. The field of ‘knowledge translation’ was still nascent; indeed, it was not until the late 1990s and early 2000s that the term ‘knowledge translation’ became widely used to describe the process of supporting the implementation of key research findings [7]. While international agencies were using health systems research to inform their policies, there was a tendency to assume that research evidence from one LMIC would be equally applicable across widely varying contexts. For example, the World Bank’s 1987 adoption of policy supporting the introduction of user fees for health services globally [8] appears to have been largely justified on the basis of studies from South East Asia demonstrating the insensitivity of populations in these locations to price changes for health services [9, 10]. Thus, while evidence was used in some quarters to support decision-making, there was little attention paid to the need for countries to have their own capacity for generating evidence, or to invest in the skills of policy-makers so that they could better understand and support research.

### Addressing the challenges

One of the most significant factors driving increasing interest in the HPSR field has been the recognition of the importance of strong health systems. This trend, described in more detail elsewhere [11], built upon the growing recognition by programmes with responsibilities for achieving the Millennium Development Goals (MDGs) that the set targets would not be achieved without better health systems. For example, as the Global Fund to Fight AIDS, Tuberculosis and Malaria and the US President’s Emergency Plan for HIV/AIDS Relief sought to scale up antiretroviral therapy, there was a rapid realisation that in sub-Saharan Africa the health workforce was inadequate to support this. Other aspects of health systems, such as drug supply, also quickly attracted attention. Similarly, the multi-country evaluation of Integrated Management of Childhood Illness [12] found that its strategy had not led to the anticipated improvements in child health, due largely to weaknesses in health systems. This recognition, along with efforts to de-mystify health systems (as in the WHO report on health systems *Everybody’s Business* [13]) were helpful in expanding interest in health systems and raising awareness about the importance of HPSR.

The MDGs targeted specific health outcomes and thus in some respects undermined a health systems approach, but from about 2008 onwards, universal health coverage (UHC) became an increasingly central rallying point for global health advocacy. UHC has obvious, direct links to HPSR, requiring an understanding of appropriate financing mechanisms not just for single diseases but for the health system as a whole, as well as knowledge on how best to organise and deliver health services so as to ensure that they are accessible, affordable and accountable.

While the ascendancy of the health systems strengthening agenda certainly paved the way for an increased focus on HPSR, others factors, notably sustained advocacy for HPSR, initiatives to clarify the field of HPSR, and efforts to better engage policy-makers and practitioners in HPSR, helped to increase interest in the field. Initially, advocacy for HPSR was scattered and uncoordinated, but the creation of the Alliance for HPSR in 1999 greatly helped to focus attention and, with strong leadership for health systems within WHO, more harmonised approaches emerged. Early publications and events, such as the Ad Hoc Committee report [14] and the 2000 World Health Report [15], prepared the ground for increased interest and investment in HPSR, but there has been more consistent advocacy since 2004, and in particular as a consequence of the Mexico Ministerial Summit [16]. The first action item in the Summit’s statement was for national governments to “*commit to fund the necessary health research to ensure vibrant health systems and reduce inequity and social injustice*”, and this was further supported by a call for research funders to “*to support a substantive and sustainable programme of health systems research aligned with priority country needs*” [16]. The Bamako ministerial meeting 4 years later provided an opportunity to take stock of progress since Mexico [17] (Table 1).

**Table 1:** Key publications and events advocating for Health Policy and Systems Research (HPSR)

Date	Publication or Event	Significance
1996	Ad Hoc Committee report “Health Policy and Systems Development: An agenda for Research” [2]	First attempt to identify global research priorities for the health policy and systems field; contributed to the establishment of the Alliance for HPSR
1997	Lejondal Meeting in Stockholm and accompanying reports and proposals [28]	This international consultative meeting at Lejondal with senior scientists, policy-makers and representatives of various agencies with a stake in HPSR led the way for an “Interim Board” for the Alliance for HPSR
2000	World Health Report: “Health Systems: Improving performance” [15]	One of the early reports to present a clear conceptual framework for health systems, and the somewhat controversial ranking of country health systems both piqued policy-maker interest and offered a set of consistent metrics for health systems
2003–04	Task Force for Health Systems Research [29]	Identified health systems research priorities to help achieve the Millenium Development Goals. Report was published in the *Lancet*
2004	Tanzania Essential Health Intervention Project (TEHIP) launch of final report “Fixing Health Systems” [30]	Said to have triggered interest in health system investments at senior levels of the Gates Foundation and the Doris Duke Charitable Trust
2004	Ministerial Summit on health research, Mexico City, Mexico [16]	Statement from the Summit called for greater investment in health systems research, but also for greater attention to the evidence-to-policy gap
2004	European Commission report on 20 years of health systems research funding [31]	Called for greater investment in HPSR, as well greater attention to capacity development for low- and middle-income partners, and getting research into policy and practice
2006	*Lancet* and Mexican Ministry of Health meeting on health system reform	Showcased national level efforts to drive policy change through HPSR
2008	Ministerial Meeting on Health Research, Bamako, Mali [17]	Follow-on from the Mexico Summit, continued to drive focus on health systems research and evidence-to-policy work, as well as assessing progress against Summit commitments
2008	High-level consultation and task force on “Scaling Up Research and Learning for Health Systems” [32]	Issued four main recommendations: (1) mobilise a high profile agenda of research and learning on health systems; (2) engage policy-makers in shaping the agenda and encourage research use; (3) strengthen country capacity for HPSR; and (4) increase financing for HPSR
2010	First Global Symposium on Health Systems Research, Montreux, Switzerland	First international conference focused on HPSR
2012	WHO Strategy on Research for Health [27]	Initiated in 2007 and developed through a consultative process, this WHO strategy document prioritised research that met health needs, and underscored investments in capacity development and knowledge translation
2012	Changing Mindsets: WHO Strategy on Health Policy and Systems Research [33]	First WHO strategy focused on HPSR
2012	Second Global Symposium on Health Systems Research, Beijing, China	Establishment of the society, Health Systems Global
2013	Research for Universal Health Coverage: World Health Report 2013 [34]	Articulates the importance of HPSR to advance progress in universal health coverage and calls for greater investment in low- and middle-income countries in HPSR
2014	Third Global Symposium on Health Systems Research, Cape Town, South Africa	
2014	Statement on Advancing Implementation Research and Delivery Science [18]	Joint statement issued by the Alliance for HPSR, USAID, WHO and the World Bank underlining the importance of implementation research
2016	Fourth Global Symposium on Health Systems Research, Vancouver, Canada	

Further support to the field of HPSR has come from the growing interest in and advocacy for the field of implementation science that culminated internationally in the 2014 Statement on Advancing Implementation Research and Delivery Science [18].

Likewise, multiple efforts have been made to help clarify the field of HPSR [19–22]. A particular challenge has been to address confusion between health systems research and health services research. During the past 15 years there has been an evolution whereby the two fields have converged considerably [3], with HPSR researchers in LMICs focusing on a more varied mix of levels of questions (macro, meso and micro) [23] and the same being true of health services researchers in high-income countries. Another issue has been the presence or absence of the “P” in HPSR. From the start of the Alliance in 1999, it was considered important to include the “P”; this was both to signal the close link between research and policy, namely the need for research to be oriented towards informing policy, and the importance of doing research not just for policy but also on policy – in other words to signal the inclusion of the fields of health policy analysis and political science. Yet, to this day, research on health decision-making is relatively neglected and health policy analysis in LMICs is still in a relatively early phase of development [24].

Finally, a key recommendations from the 2004 Mexico Summit on health research concerned promoting the greater use of evidence in policy- and decision-making. Specifically, the Summit statement called for national governments “*to establish sustainable programmes to support evidence-based public health and health care delivery systems, and evidence-based health related policies*” [16]. This call reflected growing interest globally in improved use of evidence for policy- and decision-making. The field of evidence-to-policy started in the mid-1960s and was rooted in three main domains, namely innovation diffusion, technology transfer and knowledge utilisation [25]. However, evidence-based medicine emerged as a fourth domain of importance in the mid-1980s. Globally, formal organisational structures to support evidence-based medicine were established with the creation of the Cochrane Collaboration in 1993, and the Effective Practice and Organization of Care Group, established in 1998, whose remit encompassed HPSR [26].

Growing interest in evidence-informed decision-making as a field of study, along with enhanced awareness and capacity among policy-makers and practitioners to employ evidence in policy- and decision-making, has brought the field of HPSR closer to the diverse stakeholders – policy-makers, programme managers, health system managers, health workers and civil society groups – that use evidence. This movement towards closer collaboration with research users may also have been reinforced by the calls for strengthening of national health research systems, which were integral to the statements from the Mexico Summit [16], the Bamako Ministerial Forum [17] and the WHO Strategy on Research for Health [27] . This trend towards stronger engagement of country level stakeholders was also reflected in the increased focus on implementation research [18]. Having such stakeholders more involved in identifying research priorities, and considering the implications of research, has both increased the diversity and energy in the field, and substantially added to its relevance and utility.

## Conclusions

During the past two decades, the prominence of HPSR has grown considerably. This growth is due, in good part, to a shift from disease or service-specific ways of viewing health services in LMICs towards a more integrated and systems-focused perspective, as now embodied both in UHC and in the SDGs. However, HPSR has benefitted not only from the growth of interest in health systems strengthening, but also from addressing a number of critical challenges that it faced 20 years ago.

While substantial progress has been made, there are a number of outstanding challenges, as well as opportunities, going forward. Twenty years ago, there was a greater confidence than now that ‘solutions’ to health systems challenges could be found and widely implemented. The notion that it is possible, at the global level, to define policies and strategies of more or less universal relevance would be strongly contested today. Increasingly, the need is acknowledged for rigorous comparative analyses that help understand which interventions work best in specific contexts and that fuel shared learning across countries. Further, and perhaps in part due to growing understanding of systems thinking and the relevance of complexity science to health systems research, there is greater recognition that health systems are dynamic entities. Intervention in such systems is likely to produce a counter reaction that may or may not be predictable but, regardless, is likely to require further adjustment and intervention. While this growing understanding of health systems as dynamic and adaptive may reduce the demand for universal, magic-bullet solutions, it does not mean that general policy proposals are useless, but rather that countries and sub-national jurisdictions need their own analytical capacity to trace health system changes and adapt interventions as needed.

In seeking to resolve some of the questions regarding the internal and external framing of the field of HPSR, the HPSR community has tended towards inclusivity rather than exclusivity. Analysts have argued that HPSR is defined by the questions that it seeks to address, but research within the field may assume different knowledge paradigms, and contributions may be made from different disciplinary perspectives. As a consequence, the field of HPSR has broadened. While this came from a pragmatic strategy to unify rather than fragment the field, it remains to be seen whether the field will remain cohesive over the longer term. Even within the evidence-to-policy field there is significant fragmentation – while some practitioners operate primarily within a traditional knowledge translation paradigm, others come out of the research communication field, others focus on evidence synthesis, and others are more interested in studying how evidence, among other factors, affects policy development and implementation. There have been very recent initiatives to stimulate dialogue across these groups but the process of integrating such diverse perspectives is ongoing.

Finally, we have described the parallel evolution of health services research in high-income countries and HPSR in LMICs. While the scope of these two fields now overlaps to a very high degree, there remains relatively limited engagement between researchers whose work focuses on high-income countries, and those whose work addresses LMICs. Again there have been recent efforts to bridge this divide yet, to date, such exchange has not become routine. This is a real missed opportunity, especially as countries represent a continuum of development and, at all levels of development, countries face many similar challenges, including growing burdens of non-communicable diseases, the need for more person-centred care, the rapidly increasing demands for greater health system resources, and the imperative of increasing efficiency. The fact that country responses often rely on different strategies provides a major opportunity for comparative studies in HPSR into the future.

## Abbreviations

HPSR: health policy and systems research
LMIC: low- and middle-income country
MDG: Millennium Development Goal
UHC: universal health coverage
